# Modified MXene/Holey Graphene Films for Advanced Supercapacitor Electrodes with Superior Energy Storage

**DOI:** 10.1002/advs.201800750

**Published:** 2018-08-17

**Authors:** Zhimin Fan, Youshan Wang, Zhimin Xie, Duola Wang, Yin Yuan, Hongjun Kang, Benlong Su, Zhongjun Cheng, Yuyan Liu

**Affiliations:** ^1^ MIIT Key Laboratory of Critical Materials Technology for New Energy Conversion and Storage School of Chemistry and Chemical Engineering Harbin Institute of Technology Harbin 150001 P. R. China; ^2^ National Key Laboratory of Science and Technology on Advanced Composites in Special Environments Harbin Institute of Technology Harbin 150080 P. R. China; ^3^ Center for Rubber Composite Materials and Structures Harbin Institute of Technology at Weihai Weihai 264209 P. R. China; ^4^ Natural Science Research Center Academy of Fundamental and Interdisciplinary Sciences Harbin Institute of Technology Harbin 150080 P. R. China

**Keywords:** holey graphene, modified MXene, rate capability, supercapacitors, volumetric performance

## Abstract

MXene films are attractive for advanced supercapacitor electrodes requiring high volumetric energy density due to their high redox capacitance combined with extremely high packing density. However, the self‐restacking of MXene flakes unavoidably decreases the volumetric performance, mass loading, and rate capability. Herein, a simple strategy is developed to prepare a flexible and free‐standing modified MXene/holey graphene film by filtration of the alkalized MXene and holey graphene oxide dispersions, followed by a mild annealing treatment. After terminal groups (—F/—OH) are removed, the increased proportion of Ti atoms enables more pseudocapacitive reaction. Meanwhile, the embedded holey graphene effectively prevents the self‐restacking of MXene and forms a high nanopore connectivity network, which is able to immensely accelerate the ion transport and shorten transport pathways for both ion and electron. When applied as electrode materials for supercapacitors, it can deliver an ultrahigh volumetric capacitance (1445 F cm^−3^) at 2 mV s^−1^, excellent rate capability, and high mass loading. In addition, the assembled symmetric supercapacitor demonstrates a fantastic volumetric energy density (38.6 Wh L^−1^), which is the highest value reported for MXene‐based electrodes in aqueous electrolytes. This work opens a new avenue for the further exploration of MXene materials in energy storage devices.

## 1 Introduction

In our daily life, portable electronic devices have become an indispensable consumer product. Apparently, those miniaturized energy storage devices need to store more energy in as little space as possible, which means that the gravimetric performance is not a crucial evaluation index but volumetric performance.^1^, ^2^, ^3^ It is well known that the excellent volumetric performance of supercapacitor electrode requires the gravimetric performance and packing density to simultaneously reach the optimum values. Unfortunately, there is a contradiction between the gravimetric performance and packing density for most supercapacitor electrodes, usually resulting in an inferior volumetric energy density. Interestingly, 2D materials are capable to easily assemble into a free‐standing thin film with attractive volumetric capacitance because of its relative ion‐accessible surface area and high density. Typically, 2D graphene films assembled by using the restacked graphene flakes deliver a relatively low gravimetric capacitance compared with those of 3D porous graphenes, but they can achieve a high volumetric capacitance because of the increased density.^4^ Similarly, the restacked thin film from 2D MoS_2_ nanosheets can show a superior volumetric capacitance (700 F cm^−3^) in virtue of its own high density (2.5 g cm^−3^).^5^ Very recently, a novel family of 2D Ti_3_C_2_ MXene film for supercapacitor electrode can exhibit a fantastically high volumetric capacitance of 900 F cm^−3^ due to its outstanding metallic conductivity, hydrophilic surface, and high density of 3.7 g cm^−3^.^6^ Therefore, MXene has drawn much attention in the field of portable electronic devices that require excellent volumetric performance.

Actually, MXene is a unique family of 2D early transition metal nitrides and carbides with the formula M*_n_*_+1_X*_n_*T*_x_*, where M stands for an early transition metal, X is either carbon or nitrogen, and T*_x_* represents the surface terminations, commonly prepared by selectively etching the A (ΙΙΙA or ΙVA element) atoms from the MAX precursors in aqueous acidic fluoride. In the process of etching, the A atoms between M*_n_*_+1_X*_n_* layers will be spontaneously replaced by fluorine (—F), oxygen (—O), and hydroxyl (—OH) terminal groups.^7^ Up to now, MXene has attracted sustained attention and showed competitive performance in the fields of electromagnetic interference shielding,^8^, ^9^, ^10^ catalysis,^11^, ^12^ energy storage,^13^, ^14^ reinforcement for composites,^15^ water purification,^16^, ^17^ light‐to‐heat conversion,^18^ gas separation,^19^ and multicolor cellular imaging.^20^ Specifically, MXene possesses clear superiority for energy storage electrode materials due to its highly hydrophilic surface combined with superexcellent metallic conductivity, which is hardly obtained in graphene or the majority of other known 2D materials. More interestingly, an extraordinarily higher volumetric capacitance can be obtained for the MXene due to its own pseudocapacitance behavior and impressive density.^21^ These characteristics render MXene particularly attractive for miniaturized and compact energy storage devices. Unfortunately, the self‐restacking of MXene flakes is commonly unavoidable during the process of assembling film electrodes on account of the intense van der Waals forces, leading to a low accessibility of the electrolyte ion, and thus reducing the utilization of electrochemical reactions. In addition, the mass loading has become an extremely critical performance metric for evaluating the supercapacitor electrode in the practical devices.^22^ However, rapid transport of electrolyte ions to all active sites of Ti_3_C_2_ MXene film remains a challenge, especially when the electrode areal mass loading close to the practical required levels (>10 mg cm^−2^). This can be ascribed to the MXene flakes easily tending to stack together to form an extremely dense structure, thereby impeding the ion transport pathways. To efficiently restrain the self‐restacking and decrease pore tortuosity, various molecular spacers such as MnO_2_,^23^, ^24^, ^25^ carbon nanotubes,^26^, ^27^, ^28^ and polypyrrole^29^, ^30^ have been inserted between MXene layers, improving the accessibility of electrolyte ions and increasing the accessible electroactive sites. Although the aforementioned MXene‐based composites can achieve improved gravimetric performance, their volumetric performance is inevitably reduced due to the sacrifice of density and the formed large number of open voids that would be flooded via the electrolytes, which could reduce the proportion of the active materials involved in electrochemical reaction. Furthermore, it is also possible to improve MXene's electrochemical performance by embedding porous structures between MXene layers. Lately, Gogotsi's group prepared a 3D macroporous MXene framework by incorporating polymethyl methacrylate (PMMA) spherical templates into MXene films and then sacrificing the PMMA spherical templates, achieving MXene films with increased porosity.^31^ When used as electrode materials for supercapacitor, the 3D MXene film can deliver an outstanding rate performance and ultrahigh specific capacitance, reaching the previously unmatched gravimetric performance. Nevertheless, despite creating an abundant interconnected mesopore channel, the sizes of PMMA spherical templates reach 2 µm, leading to the sacrifice of density. Therefore, those macroporous MXene films are not suitable for application in portable electronic devices that require high volumetric energy density. Interestingly, Yan et al. devised a simple strategy to prepare reduced graphene oxide (rGO)/MXene film for supercapacitor electrode with volumetric capacitance of 1040 F cm^−3^ by electrostatic self‐assembly.^32^ Due to the addition of a small amount of graphene, the densities of rGO/MXene composite films could not be significantly affected. Thus, intercalation of graphene nanosheets between MXene flakes appears to be a very effective approach to inhibit self‐restacking of MXene flakes and retain high density. However, the graphene is a completely impermeable material that does not permit any gas or small molecule to penetrate.^33^ In addition, the electrolyte ions need to pass much longer distance in rGO/MXene film to seek the broken edge of MXene flakes and graphene nanosheets, resulting in a sluggish ion transport kinetics and thus severely affecting capacitive energy storage. Recently, our previous works have reported holey graphene/conductive polymer composites with high volumetric capacitance for supercapacitor electrodes, which have shown that the holey graphene can form an unimpeded channel with other materials for efficient ion transmission because of its intrinsically excellent electrical conductivity, large effective surface areas, and much more available edge activities for electrolyte ions transport.^34^, ^35^ Therefore, the holey graphene is a potential alternative to graphene as a space interlayer to improve the electrochemical energy storage of MXene. To the best of our knowledge, the application of the composite film of holey graphene and MXene to supercapacitor electrodes or other energy storage is extremely rare. Moreover, it is worth noting that the surface terminal —F group in the MXene can severely impede electrolyte ion transmission and reduce the proportion of Ti atoms, leading to an inferior energy storage capacity, because the pseudocapacitance of Ti_3_C_2_ MXene is largely derived from the redox reaction of Ti atom,^36^ but Yan's work has not really focused on it. Therefore, it is still a challenge to obtain the MXene/holey graphene (MX‐rHGO) film with low fluorine for supercapacitor electrode with ultrahigh volumetric performance.

In this work, for the first time, a modified MXene/holey graphene film was prepared by filtration of alkalized MXene and holey graphene oxide (HGO) dispersions, followed by annealing treatment. Alkali can not only destroy the charge balance of MXene and holey graphene oxide dispersions but also cause the —F group to be converted into a —OH group. Meanwhile, the removal of most —OH groups by annealing treatment can increase the ratio of Ti atoms, which would generate more pseudocapacitive reaction. In addition, the holey graphene is employed to effectively impede the agglomeration of MXene and form a high nanopore connectivity network, which can tremendously facilitate the ion transmission and shorten electrolyte ion transport pathways. When applied as a composite film electrode for supercapacitor, it is able to exhibit ultrahigh volumetric capacitance of 1445 F cm^−3^, superior rate capability and high mass loading, indicating its promising potential for compact and miniaturized energy storage devices.

## 2 Results and Discussion

**Figure 1:**
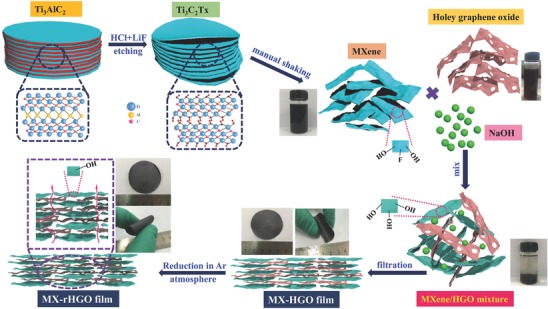
Illustration of synthesis of the modified MXene/holey graphene film.

**Figure 2:**
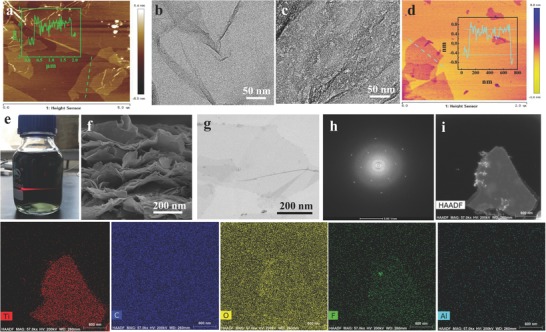
a) AFM image of the GO nanosheets. TEM images of b) GO and c) HGO. d) AFM image of the MXene flakes. e) Digital photographs of the diluted MXene colloidal solution, showing a green color and clear Tyndall scattering effect. f) SEM image of the MXene flakes. g) TEM image and h) Selected area electron diffraction (SAED) patterns of MXene, respectively. i) HAADF‐STEM image along with the EDX elemental mapping of Ti, C, O, F, and Al for MXene flakes.

The fabrication process of modified MX‐rHGO film is depicted in **Figure** **1**. Multilayered Ti_3_C_2_T*_x_* was synthesized through delaminating the Ti_3_AlC_2_ MAX phase with HCl/LiF by selectively etching the Al layers and simultaneously expanding the interlayer spacing. The as‐obtained multilayered Ti_3_C_2_T*_x_* can be facilely delaminated to monolayered flakes only by a manual shake without additional ultrasonic treatment. This is because the more surplus Li‐ions can be more effective for etching and intercalation, thus a weak force that is enough to isolate the multilayered Ti_3_C_2_T*_x_*, obtaining larger flakes with lower defects, which would possess a tremendous potential for use in electrochemical energy storage devices. Moreover, the MXene flakes are hydrophilic and negatively charged because of the existence of abundant terminating functional groups (—OH, —O, or —F); therefore, MXene colloidal solutions are able to be suspended in water by virtue of the electrostatic repulsion between neighboring flakes. Note that the HGO also has plenty of oxygen‐containing functional groups that could be very stable in water. Naturally, the electrostatic stabilization can take place in the mixture of MXene and HGO aqueous dispersions because of electrostatic repulsion and hydrophilicity between the neighboring nanosheets. When the NaOH was added into the mixture of MXene and HGO dispersion solutions, the charge balance of the colloid is destroyed due to the neutralization of the charge. As a result, large agglomerates can be seen at the bottom of the container after dozens of minutes. At the same time, the terminal —F on the surface of MXene could be easily replaced by —OH, because Ti—F bonds are extremely unstable in alkaline solution.^36^ Then, the obtained agglomerates containing a small amount of water are filtered through vacuum‐assisted filtration, and the MX‐HGO film can be easily achieved. However, it is relatively difficult to prepare a film using the MXene or HGO aqueous solutions, because a large amount of water in colloidal solutions needs to be removed. After that, the MX‐HGO film was carried out by annealing treatment at Ar atmosphere with a moderate temperature of 200 °C for 1 h to remove the vast majority of oxygen‐containing functional groups, which could significantly enhance the proportion of Ti atoms to further acquire additional pseudocapacitive reaction, finally obtaining a flexible and free‐standing MX‐rHGO film. Note that the excessive temperature can lead to the conversion of MXene's surface to nonconductive TiO_2_. The morphologies of the as‐obtained samples are shown in **Figure** **2**. The result of the atomic force microscopy (AFM) image demonstrates that the GO nanosheets have a large lateral size that exceeds several micrometers, and its average thickness is about 1 nm (Figure 2a). A uniform in‐plane pore size of a few nanometers was clearly observed in the entire basal plane of HGO (Figure 2c) while no distinct in‐plane nanopores for GO (Figure 2b), suggesting a sufficient etching of carbon atom of GO by using H_2_O_2_ monomers. The nanopores in HGO nanosheets are favorable to electrolyte ion storage and transport for electrochemical energy storage electrodes.^37^ The AFM image of MXene flakes shows flat sheet morphology, and the thickness is 1.5 nm (Figure 2d). It is worth pointing out that a green colloid with Tyndall effect can be observed in diluted dispersion of MXene (Figure 2e), demonstrating its superior hydrophilicity and dispersity. Nevertheless, high concentration of colloidal solutions of MXene optically shows black color and exhibits feeble Tyndall effect (Figure S1, Supporting Information), in good accordance with those of previous reports.^7^, ^19^ In Figure 2f, the scanning electron microscopy (SEM) image of MXene flakes shows large sizes with quite thin and clean surface that is obviously different from the Ti_3_AlC_2_ precursor (Figure S2, Supporting Information). Figure 2g shows the transmission electron microscopy (TEM) image of MXene flakes, which are extremely thin and almost transparent; the corresponding selective area electron diffraction pattern is a well‐defined hexagonal crystal symmetry (Figure 2h), in good accordance with the previously reported result.^38^ The high‐angle‐annular‐dark‐field scanning transmission electron microscopy (HAADF‐STEM) image and the corresponding energy dispersive X‐ray (EDX) elemental analysis of MXene flakes are shown in Figure 2i. Notably, the MXene flakes demonstrate a large size and clean surface, which are able to preferably stack and develop more robust structures, possessing an obvious superiority in the application of a flexible and free‐standing electrode. Moreover, it is clear that the Ti, C, O, and F elements are uniformly distributed while the Al element almost disappears, demonstrating that the Al layers have been completely removed from the Ti_3_AlC_2_ precursor. It is important to note that the HAADF‐STEM measurement of MXene flakes is to drop the dispersion on a carbon‐supported membrane; hence, we cannot visibly differentiate the distributions of C in MXene flakes. Additionally, the achieved MXene flakes by sonication of multilayer Ti_3_C_2_T*_x_* possess uneven edges accompanied with tiny dark particles (Figure S3, Supporting Information), leading to an inferior conductivity, because the MXene's surface is easily oxidized to titanium dioxide.^39^

**Figure 3:**
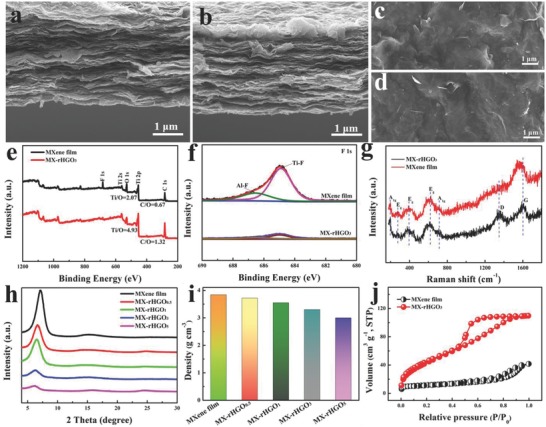
a,b) Cross‐sectional and c,d) top‐view SEM images of a,c) the pure MXene film and b,d) MX‐rHGO_3_. e) XPS spectra and f) high‐resolution F 1s spectra of MXene film and MX‐rHGO_3_, respectively. g) Raman spectra of MXene film and MX‐rHGO_3_. h) XRD patterns of the prepared MXene film and MX‐rHGO. i) The densities of the prepared MXene film and MX‐rHGO. j) N_2_ adsorption/desorption isotherms of MXene film and MX‐rHGO_3_.

**Figure** **3**a,b shows the cross‐sectional SEM image of pure MXene film and MX‐rHGO_3_, respectively. Clearly, the pure MXene film presents a well‐aligned layered and compact structure. This dense layered structure makes the pure MXene film generate excellent flexibility and mechanical properties (Figure S4, Supporting Information), but electrolyte ion transport will be extremely difficult across the entire film, specifically when increasing thickness of MXene film, resulting in an inferior rate capability. After embedding holey graphene, the obtained MX‐rHGO_3_ film can hold almost same‐layered structure without any obvious change in comparison with the pure MXene film (Figure 3b), and can still maintain its excellent flexibility (Figure S4, Supporting Information). Moreover, the MX‐rHGO_3_ film and pure MXene film show similar morphologies with abundant wrinkles from the top‐view SEM image (Figure 3c,d). The element mapping of cross‐sectional image of the pure MXene film and MX‐rHGO_3_ are displayed in Figures S5 and S6 (Supporting Information), respectively. Evidently, the Ti, C, O, and F elements distribute homogeneously in the pure MXene film. Nevertheless, the F element almost disappears and O element distribution becomes relatively weak in MX‐rHGO_3_ film, which confirms that the alkaline treatment can effectively remove most of the —F groups, and the annealing treatment is able to remove most of the oxygen‐containing groups. To further analyze the chemical compositions of the MX‐rHGO_3_ and MXene film, X‐ray photoelectron spectroscopy (XPS) was employed to test. The Ti/O ratio and C/O ratio of MXene film is 2.07 and 0.67, respectively (Figure 3e). As expected, the MX‐rHGO_3_ film possesses an enhanced Ti/O (4.93) and C/O (1.32), demonstrating that the oxygen‐containing functional groups are effectively removed during the annealing treatment. In addition, the intensity of the Ti‐F peaks is significantly reduced, which confirms the —F terminal groups are almost removed after being treated by NaOH (Figure 3f). To verify the existence state of holey graphene in the as‐obtained samples, Raman spectra are displayed (Figure 3g). The pure MXene film and MX‐rHGO_3_ reveal similar Raman spectra in the Raman shift range before 1000 cm^−1^. For MXene film, the modes at 198 and 714 cm^−1^ are A_1g_ symmetry out‐of‐plane vibrations of Ti and C atoms, respectively, and the modes at 280, 390, and 621 cm^−1^ are the E_g_ group vibrations, which include in‐plane (shear) modes of Ti, C, and the terminal group atoms.^32^ After embedding holey graphene nanosheets, the two obvious broad peaks at 1348 and 1604 cm^−1^ can be assigned to the D and G bands of graphitic carbon for the MX‐rHGO_3_ film, indicating the holey graphene exists in composite film. To inspect the change of interlayer spacing of the MX‐rHGO films compared with pure MXene film, X‐ray diffraction (XRD) tests were performed (Figure 3h). The pure MXene film shows an intense peak at around 7.2°, demonstrating that the interlayer spacing is about 1.28 nm. Apparently, the (002) diffraction peak of MX‐rHGO_3_ film shifts to 6.3° when introducing holey graphene into MXene layers, confirming the embedded holey graphene efficiently expands the interlayer spacing of MXene flakes, which would possess an enormous potential to increase ion transport channels. Furthermore, the intensities of (002) peaks decrease as the holey graphene content increases, demonstrating the reduction of the stacking order of MXene layers because of the addition of holey graphene, thus inevitably bringing about the decrease of electrical conductivity and material density. Obviously, the density and electrical conductivity of the obtained samples gradually decreased with the addition of holey graphene (Figure 3i and Figure S7, Supporting Information), because the holey graphene itself has inferior electric conductivity and low density compared with MXene. Interestingly, the MX‐rHGO_3_ film can still retain an extraordinary electrical conductivity of 1770 S cm^−1^ accompanied with a competitive density of 3.3 g cm^−3^, thereby guaranteeing a high volumetric performance in energy storage devices. Furthermore, the nitrogen adsorption–desorption measurement was employed to further verify the mitigative restacking structures of the MX‐rHGO film (Figure 3j). The MXene film merely has a specific surface area of 1.5 m^2^ g^−1^ due to the serious restacking dense structure, unavoidably impeding the approachability of MXene flakes to electrolyte ions. Amazingly, the specific surface area of MX‐rHGO_3_ film was achieved as 68 m^2^ g^−1^, which is distinctly superior to pure MXene film. This is because the holey graphene itself possesses a high surface area on account of the presence of abundant nanopores in the basal plane. In addition, the introduction of holey graphene into the MXene layers can effectively reduce its severe restack, forming a highly interconnected pore connectivity channel, which is very favorable to accelerate the transport and diffusion of electrolyte ion in energy storage applications requiring fast charge/discharge processes.

**Figure 4:**
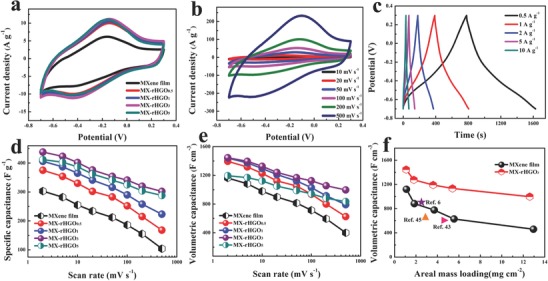
a) CV curves of MXene film and MX‐rHGO at a scan rate of 20 mV s^−1^. b) CV curves of MX‐rHGO_3_ at the different scan rates ranging from 10 to 500 mV s^−1^. c) Constant current charge–discharge curves of MX‐rHGO_3_ at different current densities. d) Gravimetric and e) volumetric capacitances of the MXene and MX‐rHGO at different scan rates. f) Effect of areal mass loading on the volumetric capacitance of MXene film and MX‐rHGO_3_ in comparison with some previous reports.

As mentioned above, the MX‐rHGO film displays a visibly increased interlayer spacing, thorough surface modification, rapid electrolyte ion accessibility, short ion/electron pathways, and high density. As a result, this competitive MX‐rHGO film should be a tremendously potential electrode material for supercapacitor and other electrochemical energy storage, which are able to acquire greatly improved high rate capability and volumetric performance. The free‐standing MX‐rHGO film could be used directly as working electrodes without additives, and the electrochemical performance was first measured in a three‐electrode system. **Figure** **4**a shows the cyclic voltammetry (CV) profiles of the pure MXene film and MX‐rHGO at a scan rate of 20 mV s^−1^. Clearly, a pair of broad redox peaks can be observed for all CV curves during the complete voltammetric cycles, demonstrating that the capacitance largely derives from the pseudocapacitance based on the sustaining and reversible redox reaction of the Ti atoms.^27^, ^32^ Notably, the MX‐rHGO_0.5_ film shows an obviously higher CV integration area than pure MXene film. This is because the introduction of holey graphene into the MXene layer can effectively increase the interlayer space and alleviate the restacking of the MXene nanosheets, thereby obtaining an increased utilizable surface area to the electrolyte ion. Additionally, the nanopores in holey graphene can tremendously facilitate the ion transport across the entire surface area and induce the holey graphene as well as MXene flakes to form a high nanopore connectivity network. More importantly, the terminal —OH groups on the surfaces of MXene are availably removed by annealing treatment, which can make more Ti atoms participate in redox reaction, accordingly getting additional pseudocapacitance in the electrochemical reaction.^36^, ^40^ When the amount of holey graphene is added into the composite film up to 3 wt%, the corresponding CV integration area reaches the maximum, demonstrating that it yields highest capacitance. Nevertheless, the MX‐rHGO_5_ film displays a lower CV integration area than MX‐rHGO_3_ film, because the large quantities of holey graphene will visibly reduce the electrical conductivity and pseudocapacitive reaction of the obtained composite films. Furthermore, it is important to note that the CV integration area of MX‐rHGO_3_ film is obviously larger than MX‐rGO_3_ film (Figure S8, Supporting Information), showing its better electrochemical properties. Meanwhile, the MX‐rHGO_3_ film can still maintain the CV profiles with an inappreciable shift of the cathodic and anodic peak even at a high scan rate (500 mV s^−1^) without obvious distortion, suggesting its highly capacitive nature with impressive ion response and superior rate capability (Figure 4b). Oppositely, the pure MXene film and MX‐rGO_3_ show a severely distorted CV curves with increasing scan rate (Figure S9, Supporting Information). Figure 4c shows the constant current charge/discharge curves of the MX‐rHGO_3_ film electrode at different current densities. It is clear that these charge/discharge curves deliver a triangular shape with an inappreciable deviation, indicating that the capacitance is the combination of double‐layer capacitance and pseudocapacitance, in good accordance with the results of the CV tests. To better know the electrochemical behavior of the achieved electrodes, Figure 4d depicts the specific capacitances of the MXene film and MX‐rHGO film at a scan rate of 2 to 500 mV s^−1^. The MX‐rHGO_3_ film shows the highest gravimetric capacitance at various scan rates. Particularly, MX‐rHGO_3_ film displays a gravimetric capacitance of 438 F g^−1^ while the pure MXene film only yields 303 F g^−1^ at 2 mV s^−1^. More importantly, the MX‐rHGO_3_ film could still retain as high as 302 F g^−1^ even at 500 mV s^−1^ with an outstanding rate capability (69%), much better than those of MX‐rHGO_0.5_ (45%) and MX‐rHGO_1_ (55%), indicating that the interconnected pore connectivity and short diffusion pathways are favorable for fast charge transport. Note that the pure MXene film electrode merely possesses a capacitance retention of 34% at 500 mV s^−1^ that is visibly less than those of MX‐rHGO film electrodes. This is because the severe self‐restacking between the MXene layers could lose the unimpeded channels, thereupon severely impeding the availability of electrolyte ions and leading to an inadequate Faraday reaction. In addition, the densities of MX‐rHGO films are merely slightly smaller than that of pure MXene film; therefore, the volumetric capacitance of MX‐rHGO_3_ film can reach up to 1445 F cm^−3^ at 2 mV s^−1^ (Figure 4e), which surpasses many known MXene‐based materials^6^, ^26^, ^30^, ^31^, ^32^, ^40^, ^41^, ^42^ and graphene/conducting polymer composites,^34^, ^35^, ^43^ and is even close to MXene hydrogel and RuO_2_/graphene supercapacitors^44^; more specific data are shown in Table S1 (Supporting Information). Moreover, the gravimetric and volumetric capacitances of MX‐rHGO_5_ film are significantly smaller than that of MX‐rHGO_3_ film due to the reduction of pseudocapacitance and packing density. This is mainly because the capacitance of Ti_3_C_2_T*_x_* MXene presents a pseudocapacitive charge storage mechanism in a sulfuric electrolyte; thus, an excessive addition of holey graphene will reduce the proportion of MXene as well as packing density. In addition, it is worth noting that the mass loading has become an extremely important parameter for evaluating the supercapacitor electrode in the practical devices, but most of the currently reported mass loadings are relatively low (<10 mg cm^−2^) because of the severe ion diffusion limitations in thick electrodes.^22^ Interestingly, the MX‐rHGO_3_ film can still retain an ultrahigh volumetric capacitance (988 F cm^−3^) when the mass loading is increased to 12.6 mg cm^−2^ (Figure 4f), which is significantly superior to many known electrode materials, such as conductive MXene “clay” (Ti_3_C_2_T*_x_* clay),^6^ compact graphene/PANI nanomolith (graphene‐PANI),^43^ and molecular stitching of grapheme film (PPD‐graphene),^45^ However, for pure MXene film, the volumetric capacitance is pronouncedly degraded with the increased mass loading owing to its sluggish ion transport kinetics.

**Figure 5:**
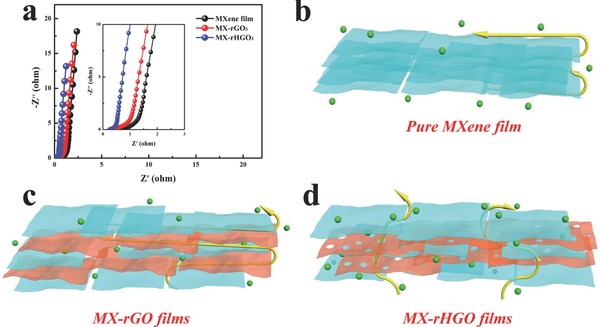
a) Nyquist plots of as‐prepared samples and the magnified high‐frequency region are shown in the inset. b–d) Schematic illustration of ion diffusion pathways across the MXene film, MX‐rGO, and MX‐rHGO, respectively.

To further illuminate the kinetics of ion transport of the MX‐rHGO films, MX‐rGO films and pure MXene film, the electrochemical impedance spectroscopy (EIS) measurements with a frequency range from 0.01 HZ to 100 kHZ were performed. As shown in **Figure** **5**a, it is clear that all of the Nyquist plots deliver similar curves containing a nearly vertical line in the low‐frequency region and an inappreciable semicircle in the high‐frequency region. Furthermore, the interfacial charge‐transfer resistance (*R*_ct_) of MX‐rHGO_3_ is evidently less than those of pure MXene film and MX‐rGO_3_, demonstrating that the ionic conductivity of MX‐rHGO_3_ film was improved after embedding holey graphene as well as subsequent annealing treatment. Additionally, the MX‐rHGO_3_ film possesses a lower diffusion resistance; thus, the slope of the plot of MX‐rHGO_3_ film is significantly greater than that of pure MXene film. The improved ion transport behavior is mainly attributed to the embedded holey graphene that provides more active sites and the removed surface group after annealing treatment further promotes ion transmission; this is because the termination groups (—F/—OH) on the surface of MXene can severely affect ion diffusion.^36^ A schematic illustration of the ion‐diffusion pathways through the pure MXene film, MX‐rGO films, and MX‐rHGO films is exhibited in Figure 5b–d, respectively. For the MXene film, the serious self‐restacking between the MXene layers can lead to the loss of the pore connectivity network, which inevitably reduced the ion‐accessible surface area and impeded electrolyte penetration. For MX‐rGO film, the self‐restacking of MXene flakes can be availably prevented due to the embedded graphene, but graphene is a completely impermeable material that does not permit any gas or small molecule to penetrate. Thus, the electrolyte ions need to pass much longer distance in MX‐rGO film to seek the broken edge of MXene flakes and graphene nanosheets, and consequently resulting in a prolonged ion transport pathway as well as slow ion transport kinetics with compromising energy density. While for MX‐rHGO film, the embedded holey graphene can efficiently expand the interlayer spacing of MXene flakes, thereby increasing the ion‐accessible surface area and creating more active sites for electrochemical reaction. In addition, it can effectively alleviate the restacking issue of the MXene nanosheets through embedding holey graphene into MXene layers, which are capable of further generating an internal open structure, improving the transmission efficiency of electrolyte ions. More importantly, the nanopores in holey graphene can tremendously facilitate the ion transport across the entire surface areas and shorten electrolyte ion transport pathways,^34^, ^37^ and induce the holey graphene and MXene flakes to form a high nanopore connectivity network. It has been proven that the critical factor determining the rate capability of pseudocapacitor electrode is the pore connectivity rather than the pore size.^35^, ^46^ This is because the high pore connectivity can accommodate the volume changes associated with fast kinetics of faradaic charge storage. Furthermore, the removed most surface termination of —OH and —F could further improve the energy storage rate. Therefore, the MX‐rHGO film electrode with highly interconnected pore connectivity channels naturally achieved an ultrahigh volumetric capacitance accompanied with impressive rate capability.

**Figure 6:**
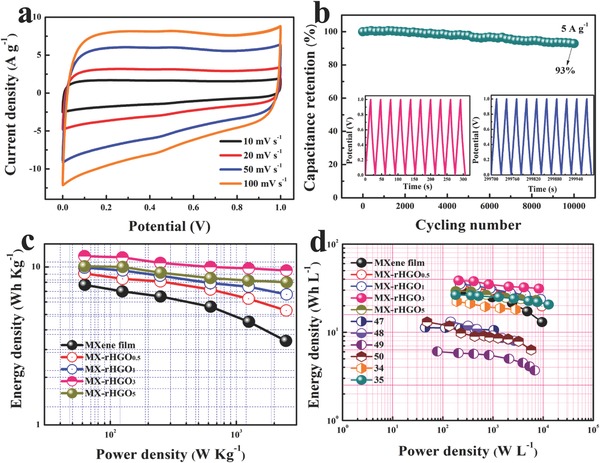
a) CV curves of the MX‐rHGO_3_‐based symmetric supercapacitor at different scan rates. b) Capacitance retention of MX‐rHGO_3_‐based symmetric supercapacitor at a current density of 5 A g^−1^ for 10 000 cycles, constant current charge–discharge curves of the initial ten cycles and last ten cycles are shown in the inset. c) Gravimetric and d) volumetric energy and power densities for MX‐rHGO_3_‐based symmetric supercapacitors in comparison with previous reports.

To further evaluate the feasibility of the MX‐rHGO film electrode in practical application, the symmetric supercapacitor was fabricated to estimate the volumetric energy density, volumetric power density, and cycling life in a 3 m H_2_SO_4_ aqueous electrolyte. It is clear that the MX‐rHGO_3_ film electrode symmetric supercapacitor delivers a nearly rectangular CV curve, and can still maintain the shape of the rectangle even at a high scan rate of 100 mV s^−1^ in the working potential window of 0–1 V (**Figure** **6**a), demonstrating its ideal capacitance behavior and excellent rate performance. The cycling life is a critical parameter for evaluating supercapacitors in practical applications. Thus, cycling performance of the MX‐rHGO_3_ film symmetric supercapacitor was conducted for 10 000 cycles at 5 A g^−1^ (Figure 6b). Amazingly, the capacitance of the supercapacitor still remains 93% of the initial capacitance, indicating its excellent long‐term cycling durability. Figure 6c shows the Ragone plots of the assembled symmetric supercapacitors, which can better demonstrate the practical operational potential of the devices. The MX‐rHGO_3_ film symmetric supercapacitor could obtain a maximum gravimetric energy density of 11.5 Wh Kg^−1^ at a power density of 62.4 W Kg^−1^, which is inferior to that of graphene, but the volumetric energy density reaches up to 38.6 Wh L^−1^ at a volumetric power density of 206 W L^−1^ and retains 31.3 Wh L^−1^ at an excellent volumetric power density of 8245 W L^−1^ due to its competitively ion transport kinetics and high density (Figure 6d), which is obviously better than those of many other known symmetric supercapacitors.^34^, ^35^, ^47^, ^48^, ^49^, ^50^ In addition, the volumetric energy density of MX‐rHGO_3_ film symmetric supercapacitor could hold a stable level with increased volumetric power density in comparison with the pure MXene film symmetric supercapacitor, demonstrating its enormous potential in capacitive energy storage.

## 3 Conclusion

In summary, we have successfully developed a facile strategy to prepare a flexible and free‐standing modified MX‐rHGO film for supercapacitor electrode with ultrahigh volumetric performance and rate capability. Because the terminal groups (—F/—OH) in MXene have almost been removed, the proportion of Ti atoms is greatly increased, which enables more pseudocapacitance. Meanwhile, the holey graphene works as a spacer to effectively prevent the self‐restacking of MXene and forms a high nanopore connectivity network, which can tremendously facilitate the ion transport and shorten electrolyte ion transport pathways. The modified MXene/holey graphene film could be used directly as a working electrode for supercapacitor without adding any additives. Due to highly interconnected nanopore connectivity accompanied with high density (3.3 g cm^−3^) and excellent electric conductivity, the MX‐rHGO film electrode can show an ultrahigh volumetric capacitance of 1445 F cm^−3^ at 2 mV s^−1^ and impressive rate capability with 69% capacitance retention at 500 mV s^−1^, and still retains an admirable volumetric capacitance (988 F cm^−3^) when the mass loading reaches up to 12.6 mg cm^−2^. Besides, the assembled symmetric supercapacitor delivers an incomparable volumetric energy density (38.6 Wh L^−1^) at a high power density of 206 W L^−1^, which is the highest value reported for MXene and carbon‐based electrodes in aqueous electrolytes. This flexible and free‐standing modified MXene/holey graphene film is an extremely promising candidate for compact and miniaturized energy storage devices. Additionally, this work proposed a novel simple strategy that can also guide future work on designing other similar materials for different applications.

## 4 Experimental Section

*Synthesis of Monolayer Ti_3_C_2_T_x_ MXene Aqueous Dispersion*: The synthesis of monolayer Ti_3_C_2_T*_x_* MXene aqueous dispersion was conducted by etching Ti_3_AlC_2_ powders with LiF/HCl based on the improved methods previously reported.^7^, ^38^ Briefly, 3.2 g of LiF (Aladin) was dissolved into 40 mL of 9 m HCl (Sinopharm Chemical Reagent Company) and kept under magnetic stirring for a few minutes. Subsequently, 2 g of Ti_3_AlC_2_ powder was slowly and carefully added into the above premixed mixture to avoid initial overheating of the solution. Then the mixture was constantly stirred at 35 °C for 24 h, and the obtained resultant was repeatedly washed with ultrapure water and centrifuged several times at 3500 rpm until the PH almost reached around neutral. Finally, the resulting homogeneous monolayer Ti_3_C_2_T*_x_* MXene can be obtained through handshaking and followed by centrifugation at 3500 rpm for 60 min.

*Synthesis of HGO*: GO was prepared using an improved Hummers method.^51^ The HGO was synthesized according to the previously reported method.^37^ Typically, 10 mL of 30 wt% H_2_O_2_ aqueous solution was dispersed into 100 mL 2 mg mL^−1^ GO dispersion for 5 h at 100 °C under stirring. Afterward, the as‐obtained HGO aqueous solution was purified by dialysis to remove the residual H_2_O_2_.

*Fabrication of Modified MX‐rHGO Film*: MX‐rHGO films were fabricated by the following procedure. Specifically, the MXene and HGO aqueous dispersions were mixed via magnetic stirring and ultrasonic treatment for 30 min to achieve MXene‐HGO composite colloidal suspensions, and the percentage of the weight of HGO in the mixture was 0.5, 1, 3, and 5, denoted as MX‐HGO_0.5_, MX‐HGO_1_, MX‐HGO_3_, and MX‐HGO_5_, respectively. Then 10 mL of 1 m NaOH aqueous was immersed in the above mixture, and then the mixture was treated by ultrasonication for 10 min. After standing 30 min, MX‐HGO was coagulated from the mixture solution. Subsequently, the obtained solid residue was purified by washing and centrifuging to remove excessive NaOH until the PH reached around neutral, and then the MX‐HGO films were obtained via vacuum filtration and drying in air at room temperature. Finally, the MX‐HGO films were carried out by annealing treatment at a low temperature of 200 °C under Ar atmosphere for 1 h to achieve MX‐rHGO films, which were labeled MX‐rHGO_0.5_, MX‐rHGO_1_, MX‐rHGO_3_, and MX‐rHGO_5_, respectively. Modified MX‐rGO films were made under identical conditions. The MXene film was prepared without the addition of NaOH as well as HGO and no calcination heat treatment for comparison.

*Characterization Methods*: The AFM images were achieved using a Bruker Dimension Fastscan in a contact and tapping mode. The morphologies of the obtained samples were examined by transmission electron microscopy (Tecnai G2 F30, FEI), scanning electron microscope (SUPRA 55 SAPPHIRE), and HAADF‐STEM (Talos F200*_x_*, FEI). XRD data were collected using Cu Ka radiation on an X‐ray diffractometer (D8 Advance, Bruker). The specific surface area was measured by using Brunauer–Emmett–Teller analyses of the N_2_ adsorption–desorption isotherms. The chemistry attributes of the obtained samples were performed by Thermo Fisher Scientific ESCALAB 250 Xi XPS.

*Electrochemical Measurements*: All electrochemical tests were measured by using a CHI 760D workstation (Shanghai Chenhua) in a 3 m H_2_SO_4_ aqueous electrolyte. A three‐electrode configuration was first used to measure the electrochemical performance of the electrode materials, in which the obtained MX‐rHGO films, Pt foil, and Ag/AgCl were used as the working, counter, and reference electrodes, respectively, and the operation potential between −0.7 and 0.3 V. To measure the real application of the as‐obtained samples, the symmetric supercapacitors were assembled by using two MX‐rHGO films with equal mass loading and nonwoven fabric (MPF30AC‐100) as the separator. The EIS was performed with a frequency range from 10 mHZ to 100 kHZ at open circuit potential of 5 mV. The gravimetric capacitance (*C*_wt_, F g^−1^) and volumetric capacitance (*C*_vol_, F cm^−3^) were calculated through the following equations

(1) $$C_{\mathrm{wt}}=\frac{1}{\Delta Vmv}\int idV$$

(2) $$C_{\mathrm{vol}}=\rho \times C_{\mathrm{wt}}$$

where *V* represents the potential windows, *m* represents the mass of electrode, *v* represents the potential scan rate, *i* is the current density, *C*_vol_ is the volumetric capacitance of the single electrode, and ρ(g cm^−3^) is the density of the electrode material. The density of electrode material is measured on the basis of the formula

(3) ρ=m/ds

where *d* is the average thickness of film that examined by SEM, *s* is the area of the film, and *m* is the mass of film. Cycling stability measurement was performed by repeating the constant current charge/discharge at 5 A g^−1^ for 10 000 cycles. The gravimetric energy density (*E*_wt_, Wh k g^−1^) and volumetric energy density (*E*_vol_, Wh L^−1^) of symmetric supercapacitor were calculated based on the following equations

(4) $$E_{\mathrm{wt}}=\int VI\mathrm{dt/}m$$

(5) $$P_{\mathrm{wt}}=E_{\mathrm{wt}}/\Delta t$$

(6) $$E_{\mathrm{vol}}=\rho E_{\mathrm{wt}}$$

(7) $$P_{\mathrm{vol}}=\rho P_{wt}$$

where *m* is the total weight of electrode materials and and *t* (s) is the discharge time.

## Conflict of Interest

The authors declare no conflict of interest.

## Supporting information

Supplementary
