# Supplementary material for: Modified MXene/Holey Graphene Films for Advanced Supercapacitor Electrodes with Superior Energy Storage
Source: Adv Sci (Weinh). 2018 Aug 17;5(10):1800750. doi: 10.1002/advs.201800750 (PMC6193160; doi:10.1002/advs.201800750)
Supplement: Supplementary file 1 — Supplementary [file ADVS-5-1800750-s001.pdf]

Copyright WILEY-VCH Verlag GmbH & Co. KGaA, 69469 Weinheim, Germany, 2018.

ADVANCED SCIENCE Open Access

Supporting Information

for *Adv. Sci.,* DOI: 10.1002/advs.201800750

Modified MXene/Holey Graphene Films for Advanced Supercapacitor Electrodes with Superior Energy Storage

*Zhimin Fan, Youshan Wang, Zhimin Xie, Duola Wang, Yin Yuan, Hongjun Kang, Benlong Su, Zhongjun Cheng, and Yuyan Liu**

Copyright WILEY-VCH Verlag GmbH & Co. KGaA, 69469 Weinheim, Germany, 2016.

Supporting Information

Modified MXene/Holey Graphene Films for Advanced Supercapacitor Electrodes with Superior Energy Storage

*By Zhimin Fan, Youshan Wang, Zhimin Xie, Duola Wang, Yin Yuan, Hongjun Kang, Benlong Su, Zhongjun Cheng and Yuyan Liu**

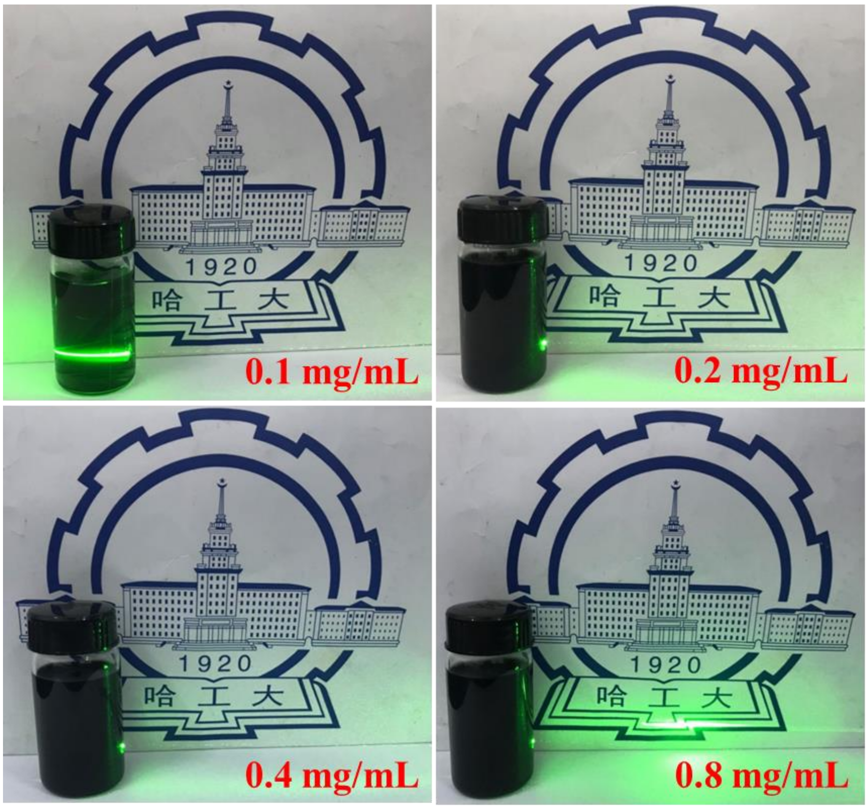

Figure S1. Tyndall effect of MXene colloidal suspension at different concentrations.

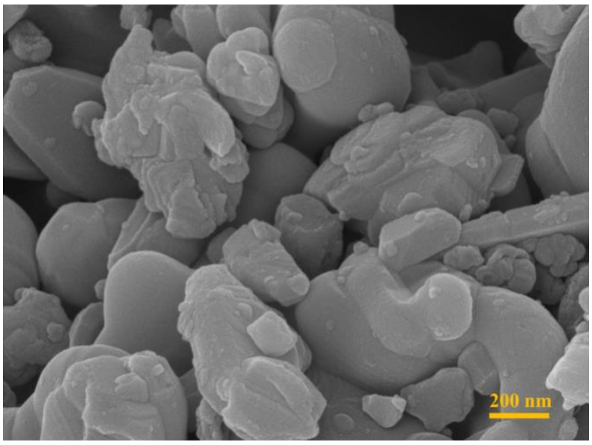

Figure S2. SEM image of Ti_3AlC_2 powder.

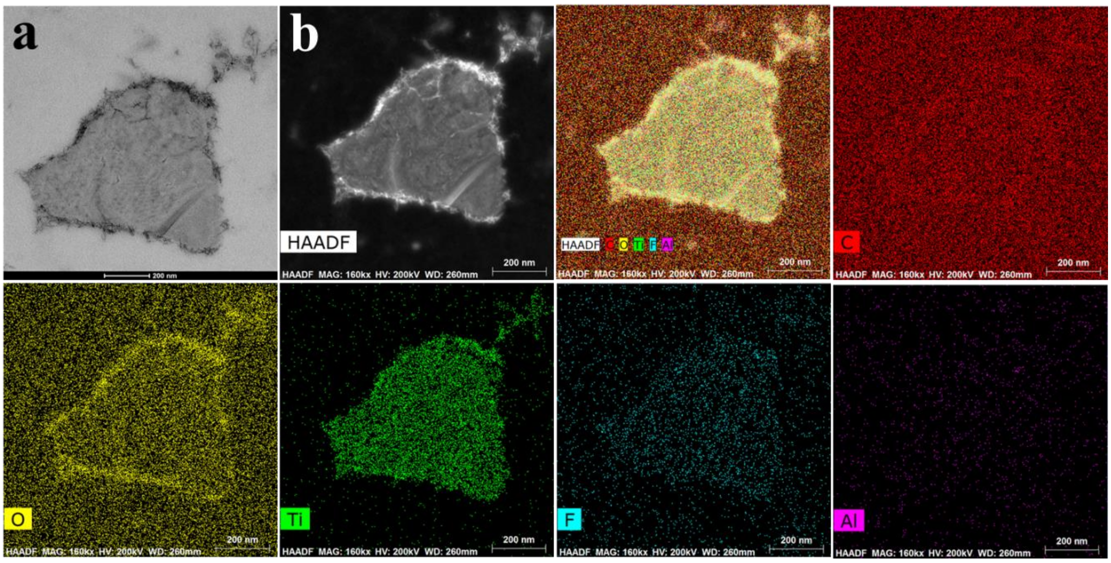

Figure S3. (a) HAADF-STEM image and (b) corresponding EDX elemental mapping of Ti, C, O, F and Al for MXene flakes produced using ultrasonic treatment.

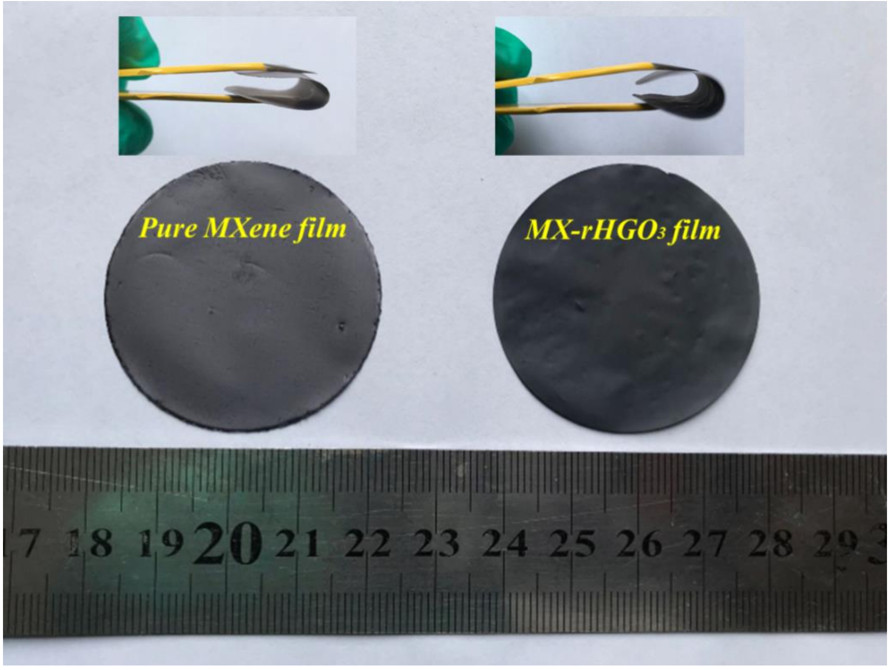

Figure S4. Photographs of MXene film and MX-rHGO$_3$ film, showing their excellent flexibility in the inset.

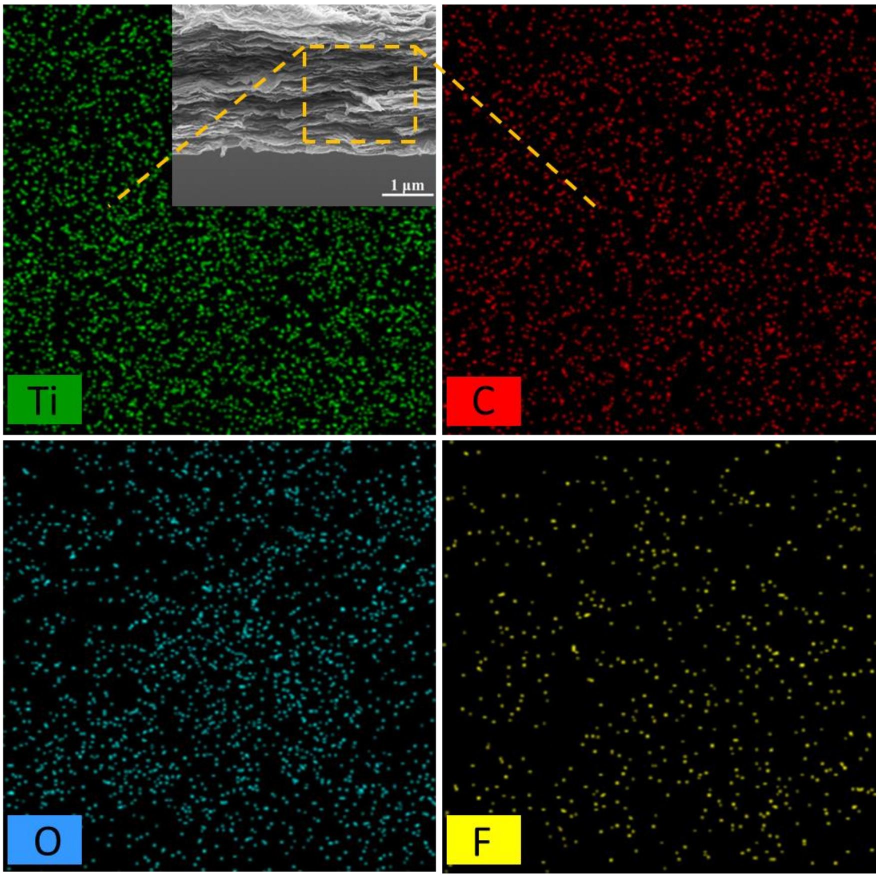

Figure S5. Cross-sectional SEM image of MXene film and corresponding elemental maps of Ti, C, O and F.

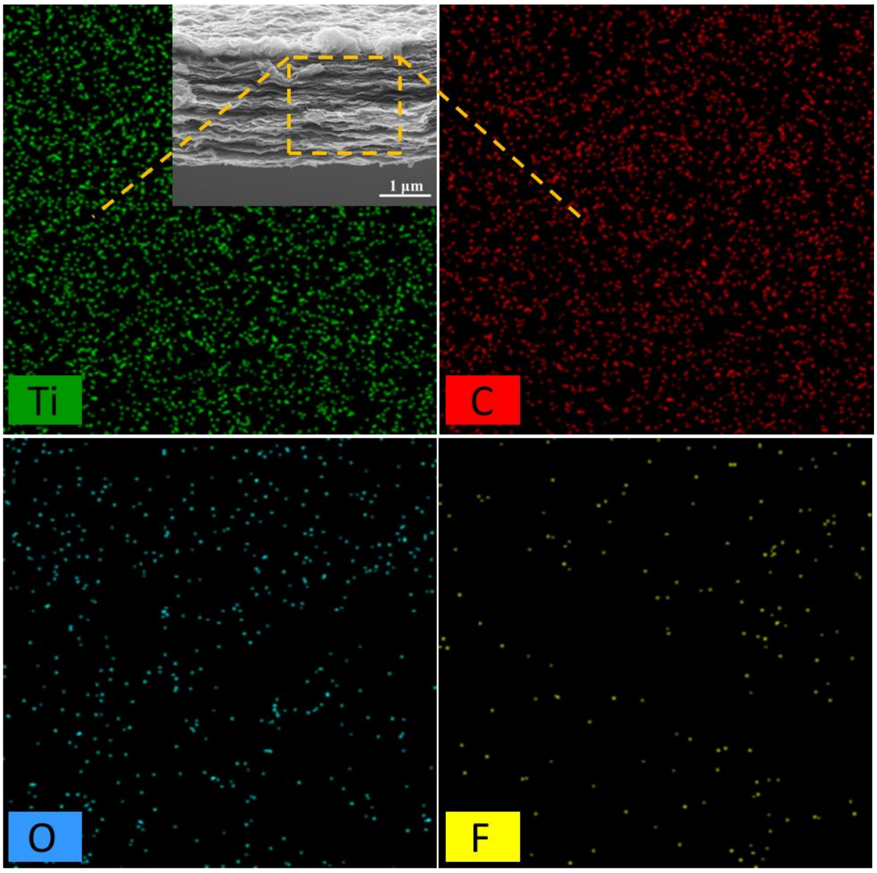

Figure S6. Cross-sectional SEM image of MX-rHGO$_3$ film and corresponding elemental maps of Ti, C, O and F.

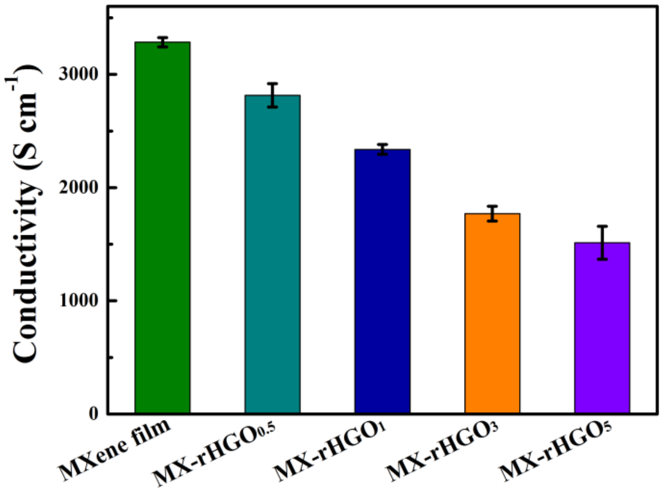

Figure S7. Electrical conductivity of the prepared MXene film and MX-rHGO.

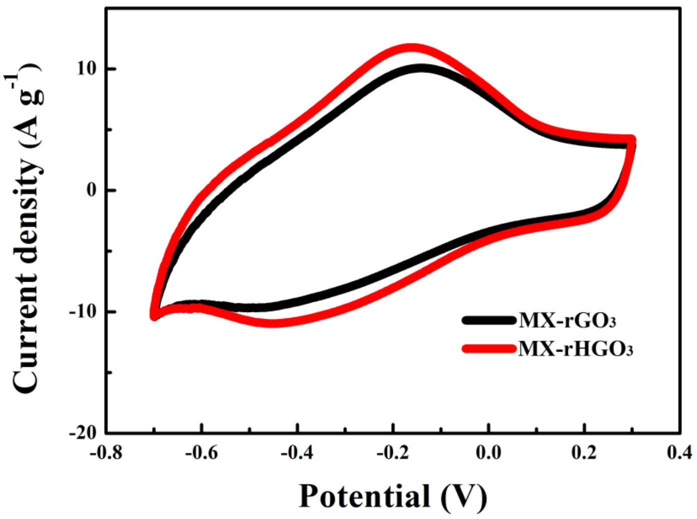

Figure S8. CV curves of MX-rGO$_3$ and MX-rHGO$_3$ at a scan rate of 20 mV s^{-1}.

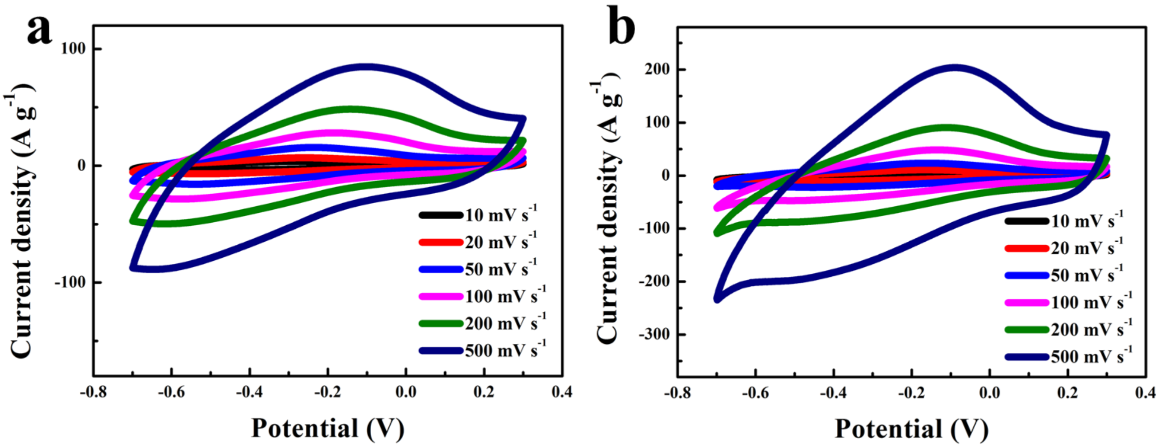

Figure S9. CV curves of (a) MXene film and (b) MX-rGO$_3$ film at the different scan rates ranging from 10 and 500 mV s^{-1}.

Table S1. Summary of volumetric capacitance of some electrodes

Electrode materials	Scan rate	C_g (F g^{-1})	C_v (Fcm^{-3})	Rate capability	Electrolyte	Refs
MXene/CNTs	2 mV s^{-1}	—	390	71.8%/200 mV s^{-1}	1M $MgSO_4$	[1]
Ultracompact MXene	2 mV s^{-1}	191.5	633	50.5%/200 mV s^{-1}	1M Li_2SO_4	[2]
MXene	2 mV s^{-1}	130	350	61.5%/100 mV s^{-1}	1M KOH	[3]
MXene/ZnO	5 mV s^{-1}	120	200	75%/100 mV s^{-1}	1M KOH	[4]
MXene/RGO	1 A g^{-1}	154.3	—	91.8%/5A g^{-1}	2M KOH	[5]
MXene/LDH	1 A g^{-1}	655	—	51%/10 A g^{-1}	6M KOH	[6]
MXene/MnO_2	5 mV s^{-1}	130	—	83%/200 mV s^{-1}	6M KOH	[7]
MXene/CNTs	5 mV s^{-1}	—	393	80%/100 mV s^{-1}	6M KOH	[8]
MXene	5 mV s^{-1}	118	—	87%/200 mV s^{-1}	6M KOH	[9]
MXene/rGO	1A g^{-1}	405	370	55.4%/10 A g^{-1}	6M KOH	[10]
HHG-PPy film	1 A g^{-1}	438	416	74%/20 A g^{-1}	6M KOH	[11]
MXene	2 mV s^{-1}	325	520	42.3%/100 mV s^{-1}	1M H_2SO_4	[12]
MXene	2 mV s^{-1}	499	226	70.1%/100 mV s^{-1}	1M H_2SO_4	[13]
MXene clay	2 mV s^{-1}	245	900	83.3%/100 mV s^{-1}	1 M H_2SO_4	[14]
HNHG/PANI slice	0.5 A g^{-1}	730	1058	84.5%/50 A g^{-1}	1 M H_2SO_4	[15]
MXene/PPy	5 mV s^{-1}	416	1000	48.1%/100 mV s^{-1}	1M H_2SO_4	[16]
RuO_2/graphene	0.1 A g^{-1}	565	1485	80%/20 A g^{-1}	1M H_2SO_4	[17]
Nanoporous MXene film	0.5 A g^{-1}	346	1142	72.5%/20 A g^{-1}	3M H_2SO_4	[18]
MXene hydrogel	2 mV s^{-1}	380	1500	—	3M H_2SO_4	[19]
MXene<br>MXene/rGO	2 mV s^{-1}	330.2<br>335.4	1221.6<br>1039	20.7%/1V s^{-1}<br>60.9%/1V s^{-1}	3M H_2SO_4	[20]
**MXene/rHGO**	2 mV s^{-1}	**438**	**1445**	**69%/500 mV s^{-1}**	**3M H_2SO_4**	**This work**

References

[1] M. Q. Zhao, C. E. Ren, Z. Ling, M. R. Lukatskaya, C. Zhang, K. L. V. Aken, M. W. Barsoum and Y. Gogotsi, *Adv. Mater*. **2015**, *27*, 339.

[2] C. Yang, Y. Tang, Y. Tian, Y. Luo, Y. He, X. Yin and W. Que, *Adv. Funct. Mater*. **2018**, *28*, 1705487.

[3] M. R. Lukatskaya, O. Mashtalir, C. E. Ren, Y. Dall'Agnese, P. Rozier, P. L. Taberna, M. Naguib, P. Simon, M. W. Barsoum and Y. Gogotsi, *Science* **2013**, *341*, 1502.

[4] F. Wang, M. Cao, Y. Qin, J. Zhu, L. Wang and Y. Tang, *RSC Adv*. **2016**, *6*, 88934.

[5] C. Zhao, Q. Wang, H. Zhang, S. Passerini and X. Qian, *ACS Appl. Mater. Interfaces* **2016**, *8*, 15661.

[6] Y. Wang, H. Dou, J. Wang, B. Ding, Y. Xu, Z. Chang and X. Hao, *J. Power Sources* **2016**, *327*, 221.

[7] Y. Tang, J. Zhu, C. Yang and F. Wang, *J. Alloys Compd*. **2016**, *685*, 194.

[8] P. Yan, R. Zhang, J. Jia, C. Wu, A. Zhou, J. Xu and X. Zhang, *J. Power Sources* **2015**, *284*, 38.

[9] J. Zhu, Y. Tang, C. Yang, F. Wang and M. Cao, *J. Electrochem. Soc.* **2016**, *163,* A785.

[10] S. Xu, G. Wei, J. Li, W. Han and Y. Gogotsi, *J. Mater. Chem. A* **2017**, *5*, 17442.

[11] Z. Fan, J. Zhu, X. Sun, Z. Cheng, Y. Liu and Y. Wang, *ACS Appl. Mater. Interfaces* **2017**, *9*, 21763.

[12] Y. Dall'Agnese, M. R. Lukatskaya, K. M. Cook, P. L. Taberna, Y. Gogotsi and P. Simon, *Electrochem. Commun*. **2014**, *48*, 118.

[13] M. Hu, Z. Li, H. Zhang, T. Hu, C. Zhang, Z. Wu and X. Wang, *Chem. Commun*. **2015**, *51*, 13531.

[14] M. Ghidiu, M. R. Lukatskaya, M. Q. Zhao, Y. Gogotsi and M. W. Barsoum, *Nature* **2014**, *516*, 78.

[15] Z. Fan, Z. Cheng, J. Feng, Z. Xie, Y. Liu and Y. Wang, *J. Mater. Chem. A* **2017**, *5,* 16689.

[16] M. Boota, B. Anasori, C. Voigt, M. Q. Zhao, M. W. Barsoum and Y. Gogotsi, *Adv. Mater*. **2016**, *28*, 1517.

[17] H. Ma, D. Kong, Y. Xu, X. Xie, Y. Tao, Z. Xiao, W. Lv, H. D. Jang, J. Huang and Q. H. Yang, *Small* **2017**, *13*, 1701026.

[18] Z. Fan, Y. Wang, Z. Xie, X. Xu, Y. Yuan, Z. Cheng and Y. Liu, *Nanoscale* **2018**, *10*, 9642.

[19] M. R. Lukatskaya, S. Kota, Z. Lin, M. Q. Zhao, N. Shpigel, M. D. Levi, J. Halim, P. L. Taberna, M. W. Barsoum, P. Simon andY. Gogotsi, *Nat. Energy* **2017**, *2*, 17105.

[20] J. Yan, C. E. Ren, K. Maleski, C. B. Hatter, B. Anasori, P. Urbankowski, A. Sarycheva and Y. Gogotsi, *Adv. Funct. Mater*. **2017**, *27*, 1701264.
